# Design and Validation of a Hand Interossei Muscle Dynamometer (HIMDNA) for Finger Abduction and Adduction Strength Measurement

**DOI:** 10.1007/s10439-026-04077-x

**Published:** 2026-03-21

**Authors:** Seung Yeon Cho, Geunwu Gimm, Sungwoo Park, Seung Jae Choi, Jaeyoung Kim, Minwoo Cho, Jihyeung Kim, Sungwan Kim

**Affiliations:** 1 Interdisciplinary Program in Bioengineering, The Graduate School, Seoul National University, 1 Gwanak-ro, Gwanak-gu, Seoul, 08826 Republic of Korea; 2 Department of Biomedical Engineering, Seoul National University College of Medicine, 103 Daehak-ro, Jongno-gu, Seoul, 03080 Republic of Korea; 3 Department of Orthopaedic Surgery, Seoul National University Hospital, 101 Daehak-ro, Jongno-gu, Seoul, 03080 Republic of Korea; 4 Institute of Convergence Medicine with Innovative Technology, Seoul National University Hospital, 101 Daehak-ro, Jongno-gu, Seoul, 03080 Republic of Korea; 5 Department of Transdisciplinary Medicine, Seoul National University Hospital, 101 Daehak-ro, Jongno-gu, Seoul, 03080 Republic of Korea; 6 Department of Clinical Medical Sciences, Seoul National University College of Medicine, 103 Daehak-ro, Jongno-gu, Seoul, 03080 Republic of Korea; 7 Department of Medicine, Seoul National University College of Medicine, 103 Daehak-ro, Jongno-gu, Seoul, 03080 Republic of Korea; 8 Institute of Medical and Biological Engineering, Seoul National University Medical Research Center, 103 Daehak-ro, Jongno-gu, Seoul, 03080 Republic of Korea

**Keywords:** Hand interossei muscles, Load-cell dynamometer, Finger abduction and adduction, Quantitative strength measurement, Ulnar neuropathy

## Abstract

**Purpose:**

Interossei muscles of the hand, innervated primarily by the ulnar nerve, are essential for coordinated finger abduction and adduction. Quantitative strength assessment of these key actions, which support precision grip and fine motor control, may aid in the diagnosis and monitoring of neuropathic conditions including cubital tunnel syndrome. This study aimed to develop and validate a novel Hand Interossei Muscle Dynamometer (HIMDNA) for quantifying finger abduction and adduction strength for the index to little fingers and to establish normative values for healthy adults.

**Methods:**

HIMDNA was designed based on hand morphology, incorporating a load cell within a fixed frame and a linear guide mechanism to ensure uniaxial, examiner-independent force measurements. The abduction and adduction strengths of 48 healthy adults aged 20–39 years were measured and validated against index and little finger abduction measurements obtained with a handheld dynamometer (HHD). The inter- and intra-rater reliabilities and overall usability of HIMDNA were then compared with those of HHD.

**Results:**

HIMDNA demonstrated excellent agreement with HHD (Pearson’s *r* = 0.927 ~ 0.967; *ICC* = 0.911 ~ 0.956) and superior inter- and intra-rater reliabilities (*ICC* = 0.963 ~ 0.983). Normative values were approximately 4.5 ~ 23.4 *N*, with greater strength in the radial than ulnar direction except for the little finger. Participants rated HIMDNA higher in usefulness, effectiveness, and overall satisfaction than HHD.

**Conclusion:**

HIMDNA provided a reliable, valid, and user-friendly way of quantifying finger abduction and adduction strengths, suggesting its potential as a standardized and clinically applicable tool for evaluating the strength of interossei muscles quantitatively in patients with ulnar nerve disorders.

**Supplementary Information:**

The online version contains supplementary material available at https://doi.org/10.1007/s10439-026-04077-x.

## Introduction

Interossei muscles of the hand generate the abduction and adduction forces required to separate and approximate the fingers, which are actions fundamental to fine motor control, coordinated manipulation, and precision grip [1–4]. These actions are produced primarily by the dorsal and palmar interossei, and assisted by the abductor digiti minimi and adductor pollicis, all of which are innervated by the deep branch of the ulnar nerve (C8–T1). Consequently, selective weakness of those muscles serves as an important clinical indicator of ulnar nerve dysfunction, including cubital tunnel syndrome, Guyon’s canal syndrome, and lower cervical radiculopathy [5–8]. Clinical tests such as Froment’s sign, Wartenberg’s sign, and Egawa’s test are based on these muscle actions and are commonly used to identify deficits associated with ulnar nerve impairment [9, 10]. However, these bedside assessments yield only binary outcomes (presence or absence of abnormality) and therefore lack the quantitative resolution needed to detect subtle or progressive changes in interossei muscle function. Accordingly, quantitative assessment of finger abduction and adduction strength is necessary, to support diagnosis, monitor disease progression, and evaluate recovery in patients with compressive neuropathies or neuromuscular disorders [11–13].

In routine clinical practice, interossei muscle strength is most often graded via manual muscle testing (MMT) on the 0 ~ 5 Medical Research Council (MRC) scale [14]. However, although MMT is quick and convenient, its resolution is limited, especially for mild weakness, and suffers from variable intra- and inter-rater reliability [15]. Thus, currently, handheld dynamometers (HHD) are being employed to provide a quantitative measure and for larger joints their objectivity can be enhanced using external stabilization such as a support stand [16, 17]. However, this approach is not easily applicable to the fingers, where the small contact area and complex positioning make such supports impractical, leaving the usability of this method still constrained by the examiner’s strength. Because the examiner must manually provide counterforce, HHD measurements are susceptible to examiner influence, and slight inconsistencies in alignment or stabilization may occur. In addition, the geometry of HHD restricts measurement to index and little finger abductions and greatly limits the capability of the device to perform middle and ring finger measurements. Similarly, standard HHD designs cannot readily accommodate adduction measurements and the geometries that they require without interference from adjacent digits. Whereas generic grip and pinch dynamometers provide quantitative and objective measurements, they largely assess extrinsic muscle groups rather than the intrinsic interossei [18, 19]. Electromyography (EMG), on the other hand, provides physiological information related to interossei muscles but, in the case of needle EMG, is rather invasive or, for surface EMG, susceptible to crosstalk and does not directly measure force, limiting its utility as a standalone measure of strength [20–22].

Several studies have attempted to overcome the limitations of the MMT and HHD. Devices such as the Rotterdam Intrinsic Hand Myometer (RIHM) and various handheld or thumb-specific dynamometers have been developed to provide more objective assessments of interossei muscle strength and found to exhibit good reliability [23–25]. However, prior RIHM studies have primarily reported measurements for selected fingers, and quantitative assessment of middle- and ring-finger abduction as well as adduction movements has not been fully evaluated [26–28]. Biomechanical systems using multi-axis force transducers have further advanced the field by investigating coordination across multiple fingers and establishing multidirectional force envelopes of the index finger; however, they are typically large, complex, and lack validation of measurement reliability or reproducibility [29, 30]. Consequently, existing methods are restricted in scope, accessibility, and validated reliability, underscoring the need for a compact and validated device capable of accurately quantifying abduction and adduction strength across all fingers.

This study aimed to develop and evaluate a Hand Interossei Muscle Dynamometer (HIMDNA), designed to provide reliable and quantitative measurements of finger abduction and adduction strength across all fingers. Its validity and reliability were evaluated through cross-validation against a conventional HHD in terms of index and little finger abduction measurements, in conjugation with inter- and intra-rater reliability analyses and user experience surveys. In addition, this study also sought to establish normative reference values for finger abduction and adduction strength in healthy young adults.

## Methods

### Study Subjects

Forty-eight healthy adults between the ages of 20 and 39 years were recruited from August to September 2025 for this study. Exclusion criteria included a history of hand surgery, recent trauma to the upper extremities, and other neurological conditions that might affect hand function. All participants provided written informed consent prior to participation. This study was approved by the Institutional Review Board of Seoul National University Hospital (IRB No. 2409-149-1578), and the procedures complied with the principles of the Declaration of Helsinki.

### Anthropometry

The demographics considered included age, sex, body weight, and hand dominance. Forearm circumference was measured bilaterally at the largest girth using a flexible anthropometric tape. Grip strength was assessed for both hands using an electronic handgrip dynamometer (PGF-1000, Prospecs, South Korea), while the participants stood in an upright posture with their arms relaxed and extended downward alongside the body. Pinch strength was evaluated bilaterally using a hydraulic pinch gauge (Baseline Pinch Gauge-Hydraulic-LiTE; Fabrication Enterprises, Inc., NY, USA), including the tip, key, and tripod pinch.

### HIMDNA: Hand Interossei Muscle Dynamometer

A novel HIMDNA was designed to quantify the isometric strengths of the index, middle, ring, and little fingers in both abduction and adduction directions. The main structure was manufactured from aluminum alloy 6061 and finished with black anodization to provide mechanical rigidity, light weight, and surface durability.

**Fig. 1:**
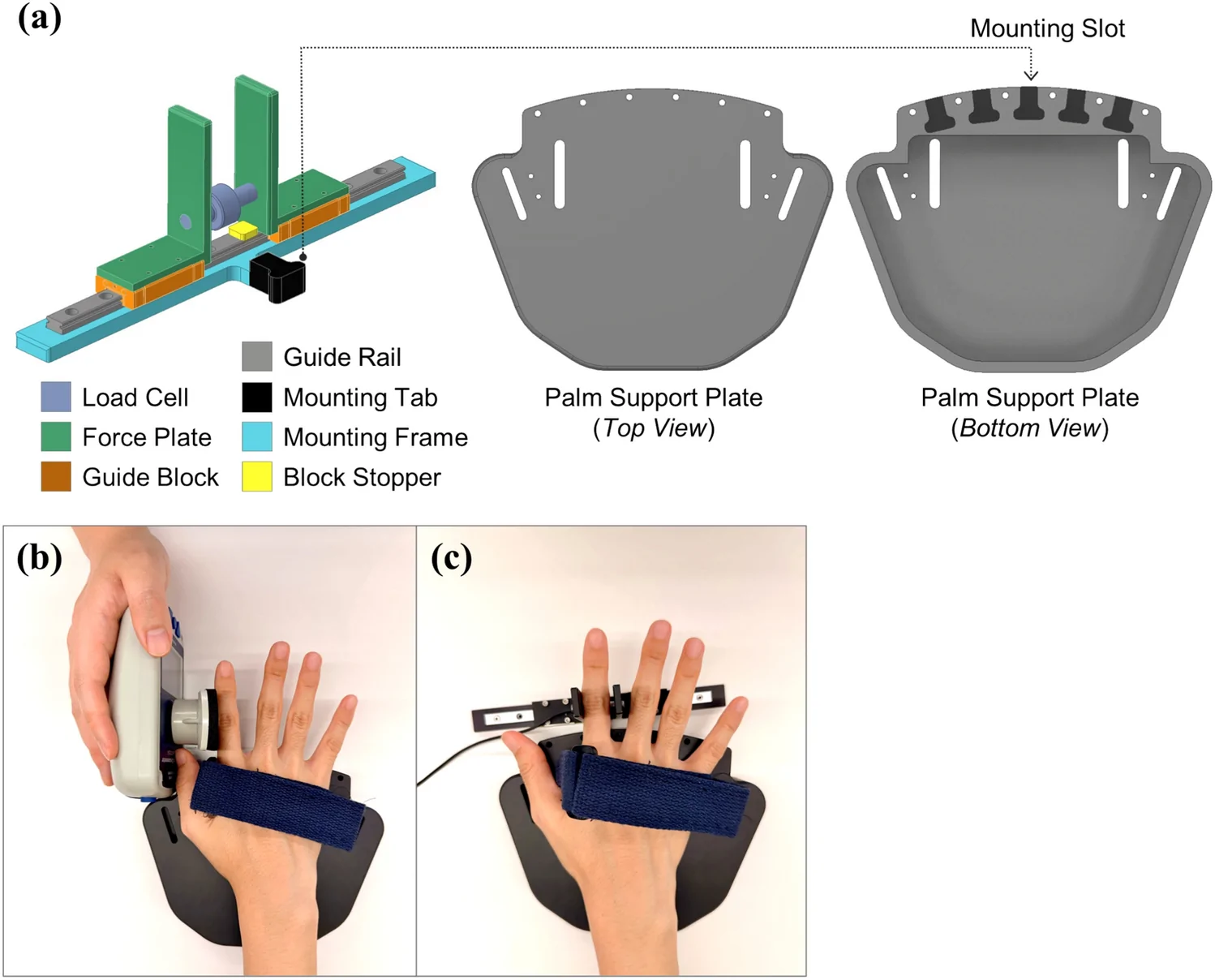
**a** Design components and configuration: the system consists of a palm support plate and load cell (blue) mounted between two force plates (green) attached to linear guide blocks (orange) sliding along the guide rail (gray); the assembly is supported by a mounting frame (sky blue) and a stopper (yellow) secured on one side to enable isometric measurements in the other direction. Examples of measurement positions for index finger assessment with **b** HHD and **c** HIMDNA

The hardware configuration consisted of three major components: a palm support plate, linear guide rail, and sensor-embedded block assembly (Fig. 1). The palm plate was contoured according to the average hand dimensions of young adults [31, 32], allowing stable placement of the hand while isolating finger movements. A strap was used to secure the dorsum of the hand against the plate to minimize vertical or rotational displacement during measurement.

A precision linear guide (E-GMLG7H-150, MISUMI Corporation, South Korea) was installed along the anterior margin of the plate. A sliding guide block (E-GMLGB7H, MISUMI, South Korea) supported the two plates with a miniature tension/compression load cell (SMNT-13C, CASSCALE, South Korea; capacity: 20 kg) housed between them. Together with the guide rail and mounting frame, this assembly constrained the loading to the axis of the load cell or guide rail and minimized off-axis components. The mounting tab, mounting slot, and block stopper (Fig. 1a) mechanically constrain the finger movement during the measurement test, thereby enabling isometric force measurement. More importantly, the palm support plate was designed with a curved contour along its anterior edge, and multiple mounting slots were distributed along this contour (Fig. 1). The linear guide and a sensor block could be inserted into one of these slots, allowing the load cell unit to be mounted at the optimal position for each finger. This modular adjustability ensured that the proximal interphalangeal (PIP) joint of the tested finger consistently contacted the plate at a standardized location, allowing precise alignment with the target finger.

**Fig. 2:**
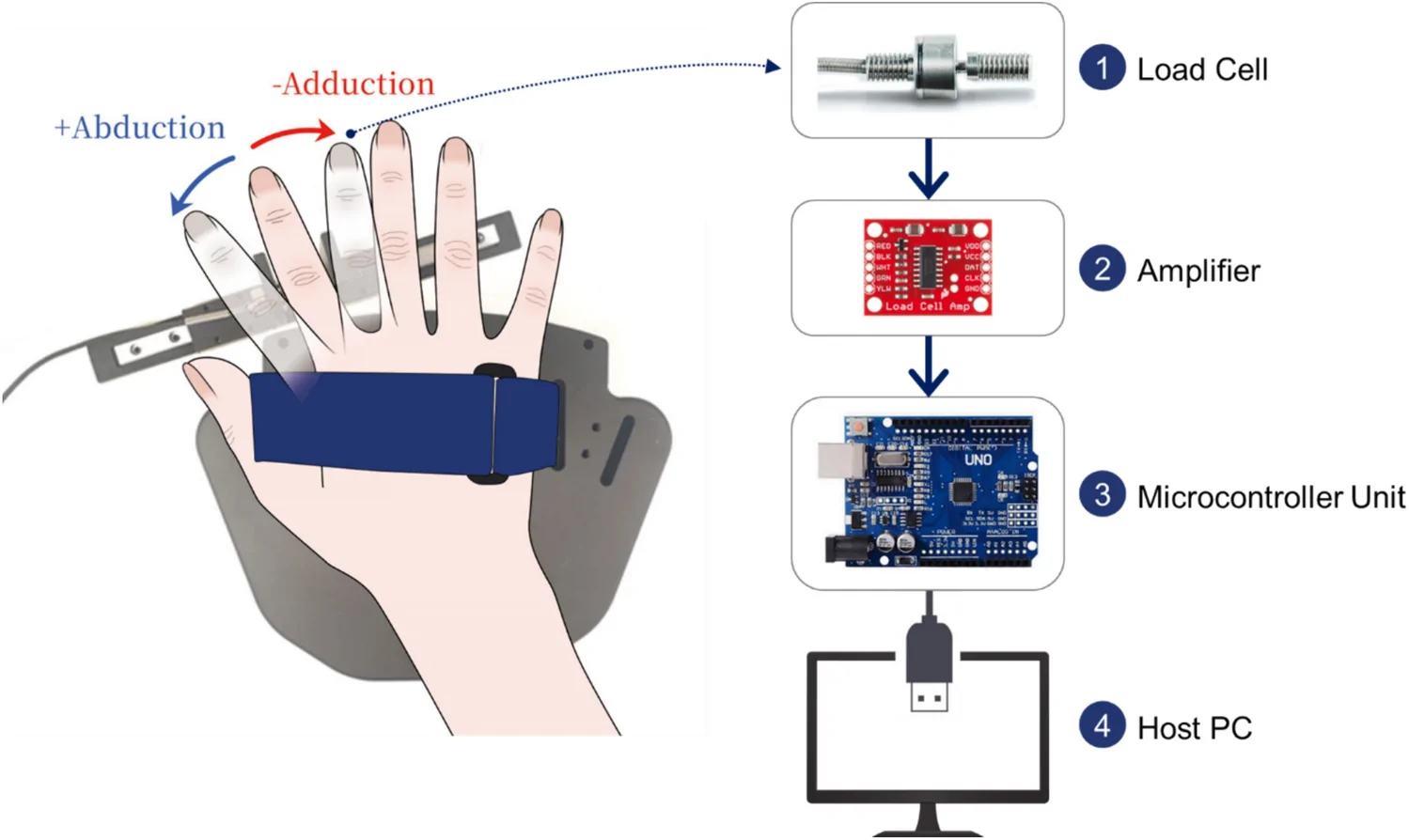
Visualization of HIMDNA during operation; Finger abduction (positive direction), and adduction (negative direction) are measured using the system composed of (1) a load cell for force measurement, (2) a load cell amplifier for signal conditioning, (3) a microcontroller (Arduino Uno) for analog-to-digital conversion and serial communication, and (4) a computer interface for real-time data acquisition and visualization

A visualization of the overall system flow is shown in Fig. 2. The load cell output signal was routed through an HX711 24-bit analog-to-digital converter (ADC) amplifier, which provided high-resolution digitization of the analog strain-gauge signals. The amplified signals were processed by an Arduino Uno microcontroller board operating at a sampling frequency of 10 Hz (Arduino IDE 2.3.4), and the data were transmitted to a host computer via serial communication at a baud rate of 9600 b/s. Based on the rated capacity and conversion depth, the effective resolution of the system was estimated to be approximately 0.02 *N*, allowing detection of small variations in intrinsic finger force. A custom MATLAB (MathWorks, Natick, MA, USA) graphical user interface (GUI) was implemented to visualize real-time force–time curves, perform baseline correction, extract peak force, and store the results in a standardized format for analysis.

Prior to conducting the tests in participants, the load cell was calibrated using a series of M1 class test weights ranging from 0.05 to 5 kg. The applied masses were converted to force (*N*) and compared with the corresponding output of the system to establish the relationship between sensor output and applied force (See Supplementary Fig. S1).

### Measurement of Maximal Abduction and Adduction Strength of the Fingers

All the strength measurements were performed in a controlled laboratory environment. Each participant was seated comfortably on a chair with the shoulder at 30° abduction, elbow at 90° flexion, and forearm at 60° pronation, resting on the table surface. An example of the position for index finger assessment is shown in Fig. 1b, c. The palm was placed on the support plate of the HIMDNA, and a strap was placed across the dorsum to secure it firmly against the plate and minimize vertical or rotational displacement during measurement. Each target finger was positioned such that the PIP joint contacted the inner surfaces of the two opposing plates integrated with the load cell. When the finger was pressed laterally toward one plate, the system recorded the force in one direction, i.e., abduction or adduction, depending on the tested hand and finger; when pressed toward the opposite plate, the force in the reverse direction was measured. This configuration allowed quantification of both abduction and adduction without repositioning any parts of the device. In this study, finger abduction and adduction were defined relative to the middle finger axis, with movement toward the middle finger considered adduction and movement away considered abduction. Accordingly, index finger adduction corresponds to ulnar deviation and ring finger adduction to radial deviation.

The participants performed approximately 3-second maximal voluntary isometric abduction and adduction trials. To obtain the maximal strength, three repetitions per direction were performed for each finger, from the index to the little, and the highest value for each was recorded for analysis. If the final repetition exceeded the preceding values, additional trials were conducted until the last measurement was no longer the highest. Only one representative value was used per subject for each finger and motion direction to avoid overrepresenting repeated measurements from individual participants. Sustained verbal encouragement was provided throughout the testing. Two trained examiners performed the measurements independently. One examiner was an orthopedic surgeon with clinical experience in musculoskeletal assessment, and the other was an engineer from the device development team. The participants underwent two repeated administrations of the same protocol to assess intra-rater reliability. To minimize muscle fatigue and potential carry-over effects, a rest interval of 5 min was provided when switching examiners, and a minimum of 5 minutes was allowed before repeating an identical session [33].

For comparative purposes, the abduction strengths of the index and little fingers were also evaluated using a conventional HHD (Model 01163, Lafayette Instrument Company, USA), which is widely used and readily accessible in clinical environments and dynamometry studies [17, 34–36]. This HHD has a reported measurement range of 0 to 300 *lbs* (1335 *N*) with an accuracy of ± 1% over full scale or ± 0.2 *lbs*, according to the manufacturer. For the index finger, the HHD pad was applied to the radial side of the PIP joint, and for the little finger, it was applied to the ulnar side of the PIP joint. The examiner manually stabilized the device at the contact site during force generation. Adduction was not assessed with the HHD because the device cannot be reliably positioned between adjacent fingers to apply directed forces in such a narrow space. Testing was conducted under the same seated posture and protocol described earlier.

### User Experience and Experience Evaluation

After completing all dynamometer assessments, the participants completed two questionnaires for each device to evaluate user acceptance and user experience: the Van der Laan Acceptance Scale and the User Experience Questionnaire short form (UEQ-S). The Van der Laan Acceptance Scale is a validated acceptance instrument comprising nine bipolar semantic-differential items grouped into two dimensions (usefulness and satisfaction), rated on a 5-point scale from − 2 to + 2; higher scores indicate greater acceptance [37]. On the other hand, the UEQ-S is a reliable and valid tool for rapid assessment of users’ feelings, impressions, and attitudes toward a product; it consists of eight items rated on a 7-point Likert scale from − 3 (very negative) to + 3 (very positive) [38, 39]. Participants were instructed to complete the questionnaires based on their overall subjective experience with each device after testing, and no additional explanations for each metric were provided to avoid response bias.

### Statistical Analysis

Descriptive statistics were calculated for the demographic, anthropometric, and strength variables. Normality of data distribution was examined using the Shapiro–Wilk test. To validate the agreement between HIMDNA and HHD, intraclass correlation coefficient (*ICC*), Pearson’s correlation coefficient (*r*), and Bland–Altman analyses were performed, including estimation of bias and 95% limits of agreement. Inter-rater reliability was assessed using ICC(2,1) (two-way random effects), which is appropriate for generalizing reliability across different examiners. Intra-rater reliability was assessed using ICC(3,1) (two-way mixed effects), a model that is more suitable for repeated evaluations by the same examiner. In addition to the ICC values, the Pearson’s *r* were calculated to illustrate the linear association between repeated measures. Reliability was considered poor for ICC values less than 0.50, moderate for 0.50 to 0.75, good for 0.75 to 0.90, and excellent for values greater than 0.90 [40]. Correlation was considered moderate for *r* values between 0.40 and 0.70, strong for 0.70 to 0.90, and very strong for values exceeding 0.90 [41]. Fisher’s z transformation and *z* tests were used to compare inter- and intra-rater ICCs and *r* values between HIMDNA and HHD. Measurement precision was further quantified by the standard error of measurement (*SEM*), calculated as *SEM* = *SD* × √(1–*ICC*), where *SD* is the pooled standard deviation of the measurements. The minimal detectable change (*MDC*) at the 95% confidence level was computed from the *SEM* using the formula: *MDC* = 1.96 × √2 × *SEM*, which indicates the smallest change that can be interpreted as a true difference beyond measurement error. Error indices, including the mean absolute error (MAE), root mean square error (RMSE), and mean absolute percentage error (MAPE), were also calculated to quantify the absolute differences across the devices. Potential outliers were examined using Grubbs’ test (two-sided, *α* = 0.05) applied to the paired differences between HIMDNA and HHD measurements. All analyses were conducted in Python (version 3.12.1) using the SciPy (version 1.13.0) and Pingouin (version 0.5.5) libraries [42, 43]. Statistical significance was set at *p* < 0.05.

## Results

### Baseline Characteristics

A total of 48 healthy adults participated in this study. Their baseline characteristics are summarized in Table 1. Based on measurements of the right side, forearm circumference was significantly greater in males (26.68 ± 1.89 cm) than in females (22.02 ± 1.99 cm; *p* < 0.001). Grip strength was also significantly higher in males (41.04 ± 8.78 kg) compared with in females (25.61 ± 3.71 kg; *p* < 0.001). Similarly, key pinch strength, which is mediated primarily by the ulnar-innervated adductor pollicis and first dorsal interosseous muscles, was greater in men (9.73 ± 1.73 kg) than in women (6.79 ± 1.50 kg).

**Table 1:** Baseline characteristics

Characteristic	Male	Female	*p*-value^a^
N, total	21	27	
Age group, 20s	11	14	
Age group, 30s	10	13	
Age, years	29.48 (3.80)	28.96 (4.24)	
Height, cm	176.10 (7.40)	163.00 (4.34)	
Weight, kg	73.11 (11.00)	57.58 (8.97)	
BMI, kg/m^2^	23.45 (2.34)	21.71 (3.60)	
Dominant hand			
Right	19 (90.5%)	24 (88.9%)	
Forearm circumference, cm			
Right	26.68 (1.89)	22.02 (1.99)	< 0.001*
Left	25.99 (1.93)	21.98 (2.03)	< 0.001*
Grip strength, kg			
Right	41.04 (8.78)	25.61 (3.71)	< 0.001*
Left	38.93 (7.93)	25.07 (4.18)	< 0.001*
Tip pinch strength, kg			
Right	7.33 (2.12)	5.20 (1.60)	< 0.001*
Left	6.65 (1.49)	5.06 (1.68)	0.001*
Key pinch strength, kg			
Right	9.73 (1.73)	6.79 (1.50)	< 0.001*
Left	9.13 (1.51)	6.60 (1.17)	< 0.001*
Tripod pinch strength, kg			
Right	9.25 (2.27)	6.95 (2.10)	0.001*
Left	9.06 (2.25)	6.98 (2.25)	0.003*

Variables are presented as mean (SD) or N (%)

^a^Comparison between male and female using Student’s *t* test

^*^Statistically significant difference (*p* < 0.05)

### Abduction and Adduction Strength of Fingers Measured with HIMDNA and HHD

The abduction and adduction strengths of the index to little fingers in the healthy young adults measured using HIMDNA and HHD are summarized in Table 2. The values ranged from 4.5 to 16.1 *N* in females and 6.9 to 24.5 *N* in males. Among the assessed motions, index finger abduction was the strongest (approximately 24 *N* in males and 16 *N* in females), whereas ring-finger abduction was the weakest (approximately 6.9 *N* in males and 4.5 *N* in females). With the exception of the little finger, the forces were generally greater in the radial direction than in the ulnar direction. Index finger adduction (approximately 12 *N* in males and 7 *N* in females) and ring finger adduction (approximately 12 *N* in males and 7 *N* in females) exhibited similar magnitudes.

**Table 2:** Abduction and adduction strengths of the index- to little-fingers in healthy young adults measured using HIMDNA and HHD, and their associations with grip and pinch strength

			Strength, *N*			Pearson’s* r*			
			Male, mean (SD)	Female, mean (SD)		Grip strength	Tip pinch strength	Key pinch strength	Tripod pinch strength
HHD	Index	ABD	24.5 (6.6)	16.1 (3.7)		0.691*	0.571*	0.550*	0.467*
HIMDNA		ABD	23.4 (6.4)	15.3 (3.8)		0.674*	0.575*	0.537*	0.458*
		ADD	12.1 (4.6)	7.0 (2.0)		0.652*	0.439*	0.535*	0.388*
	Middle	Radial ABD	15.9 (4.6)	10.3 (3.3)		0.618*	0.485*	0.539*	0.406*
		Ulnar ABD	10.8 (4.4)	6.7 (2.5)		0.628*	0.458*	0.510*	0.386*
	Ring	ADD	12.0 (4.8)	7.5 (2.8)		0.641*	0.408*	0.449*	0.417*
		ABD	6.9 (2.4)	4.5 (1.3)		0.569*	0.407*	0.358*	0.380*
	Little	ADD	9.7 (3.5)	6.0 (2.1)		0.624*	0.494*	0.403*	0.459*
		ABD	12.4 (2.6)	8.5 (2.4)		0.715*	0.608*	0.644*	0.494*
HHD		ABD	12.9 (2.5)	9.2 (2.3)		0.700*	0.613*	0.622*	0.526*

*N*, Newton;* HIMDNA,* Hand interossei muscle dynamometer; *HHD,* Handheld dynamometer; *ABD,* Abduction; *ADD,* Adduction; *SD,* Standard deviation

^*^Statistically significant correlation (*p*-value < 0.001, Pearson’s *r*)

The abduction and adduction measurements showed statistically significant moderate correlations with conventional hand strength metrics, including grip, tip pinch, key pinch, and tripod pinch (*r* = 0.358 ~ 0.715; *p* < 0.001) (Table 2). Little finger abduction showed the strongest correlations with the conventional metrics, particularly with grip (HHD *r* = 0.700; HIMDNA *r* = 0.715) and key pinch (HHD *r* = 0.622; HIMDNA *r* = 0.644). The second strongest correlations were observed for index finger abduction. By contrast, ring finger abduction showed the lowest correlations with the conventional metrics (*r* = 0.358 ~ 0.569).

### Validation of HIMDNA Against HHD

**Fig. 3:**
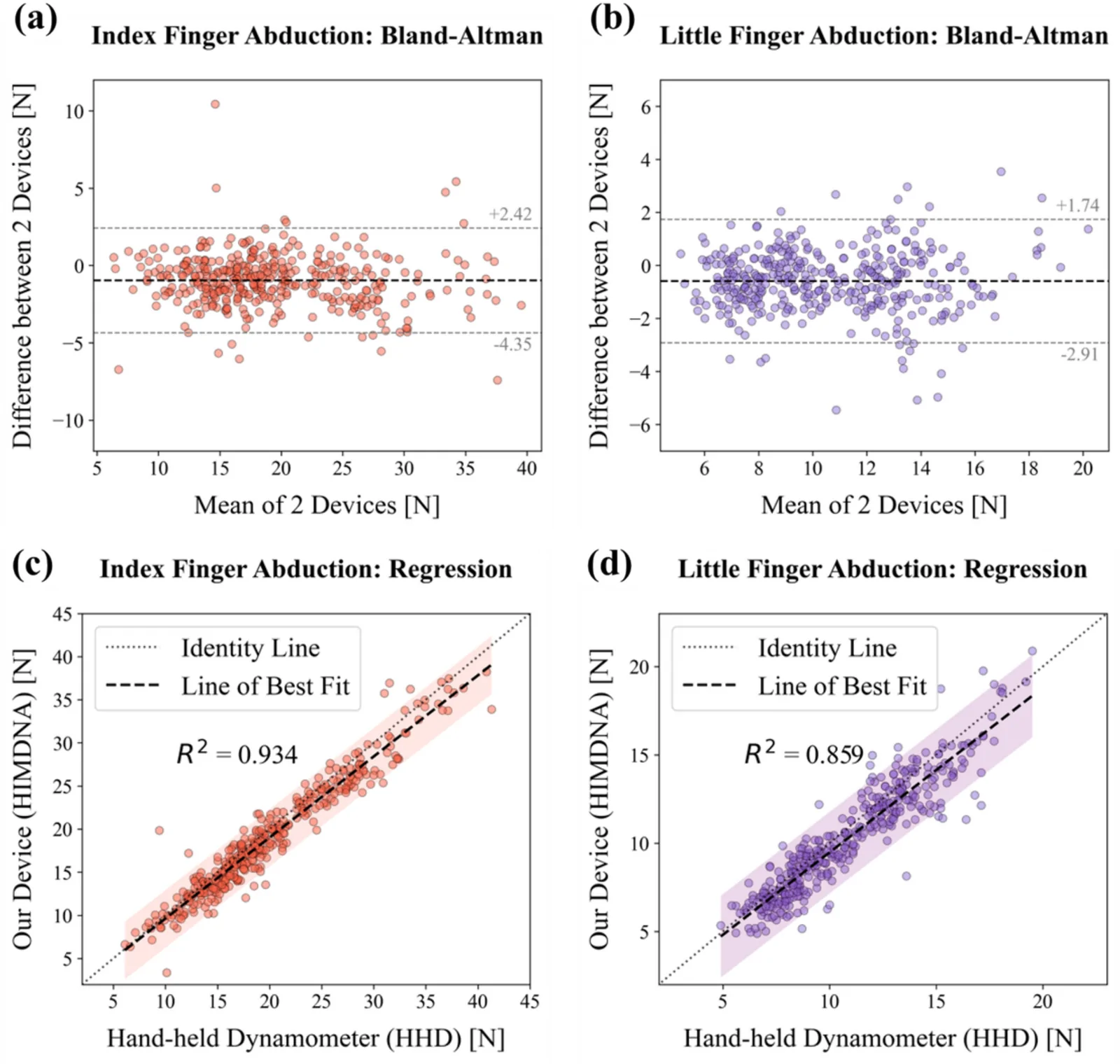
Bland–Altman analyses of agreement between HIMDNA and HHD for **a** index finger abduction and **b** little finger abduction measurements; linear regression analyses of agreement between HIMDNA and HHD for **c** index finger abduction and **d** little finger abduction measurements

The agreement between HIMDNA and HHD in assessing the abduction strengths of the index and little fingers is summarized in Table 3 and Fig. 3. The inter-device reliability was excellent, with ICC values of 0.956 for the index finger and 0.911 for the little finger, and the corresponding correlations were very strong (*r* = 0.967 and 0.927, respectively). Although HIMDNA consistently produced slightly lower values than did HHD, as indicated by Bland–Altman biases of − 0.967 (95% CI, − 1.140 to − 0.793) for the index finger and − 0.587 (95% CI, − 0.707 to − 0.468) for the little finger, the magnitudes of these biases were minimal and not clinically meaningful. Meanwhile, the MAPE between the two devices was 8.00% for the index finger and 9.51% for the little finger.

**Table 3:** Agreement between HIMDNA and HHD for index- and little-finger abduction strength

Finger	ICC_(2,1)_	Pearson’s *r* (*p*-value)	Bland–Altman			Error		
			Bias (95% CI)	SD of diff.	95% LoA	MAE [N]	RMSE [N]	MAPE [%]
Index	0.956	0.967 (< 0.001)	– 0.967 (– 1.140, – 0.793)	1.726	– 4.349, 2.416	1.492	1.976	8.00
Little	0.911	0.927 (< 0.001)	– 0.587 (– 0.707, – 0.468)	1.187	– 2.914, 1.739	1.012	1.323	9.51

*HIMDNA,* Hand interossei muscle dynamometer; *HHD,* Handheld dynamometer; *ICC*, Intraclass correlation coefficient; *SD* of diff., Standard deviation of the difference; LoA, Limits of agreement; *MAE,* Mean absolute error; *RMSE,* Root mean square error; *MAPE,* Mean absolute percentage error

### Reliability Assessment of HIMDNA and Comparison with HHD

**Fig. 4:**
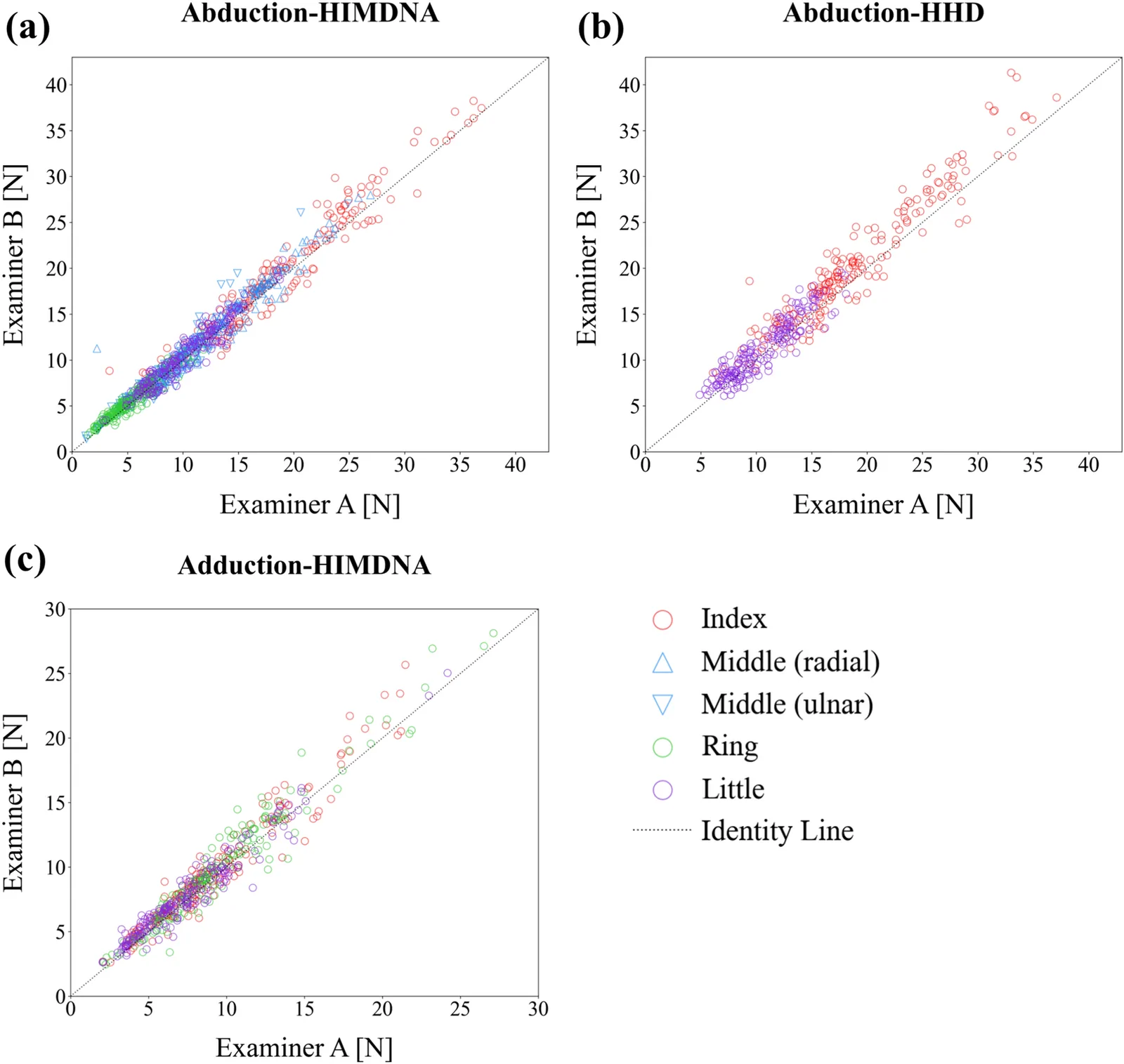
Linear regression analyses of inter-rater reliability in abduction measurements obtained with **a** HIMDNA and **b** HHD and **c** adduction measurements obtained with HIMDNA

**Fig. 5:**
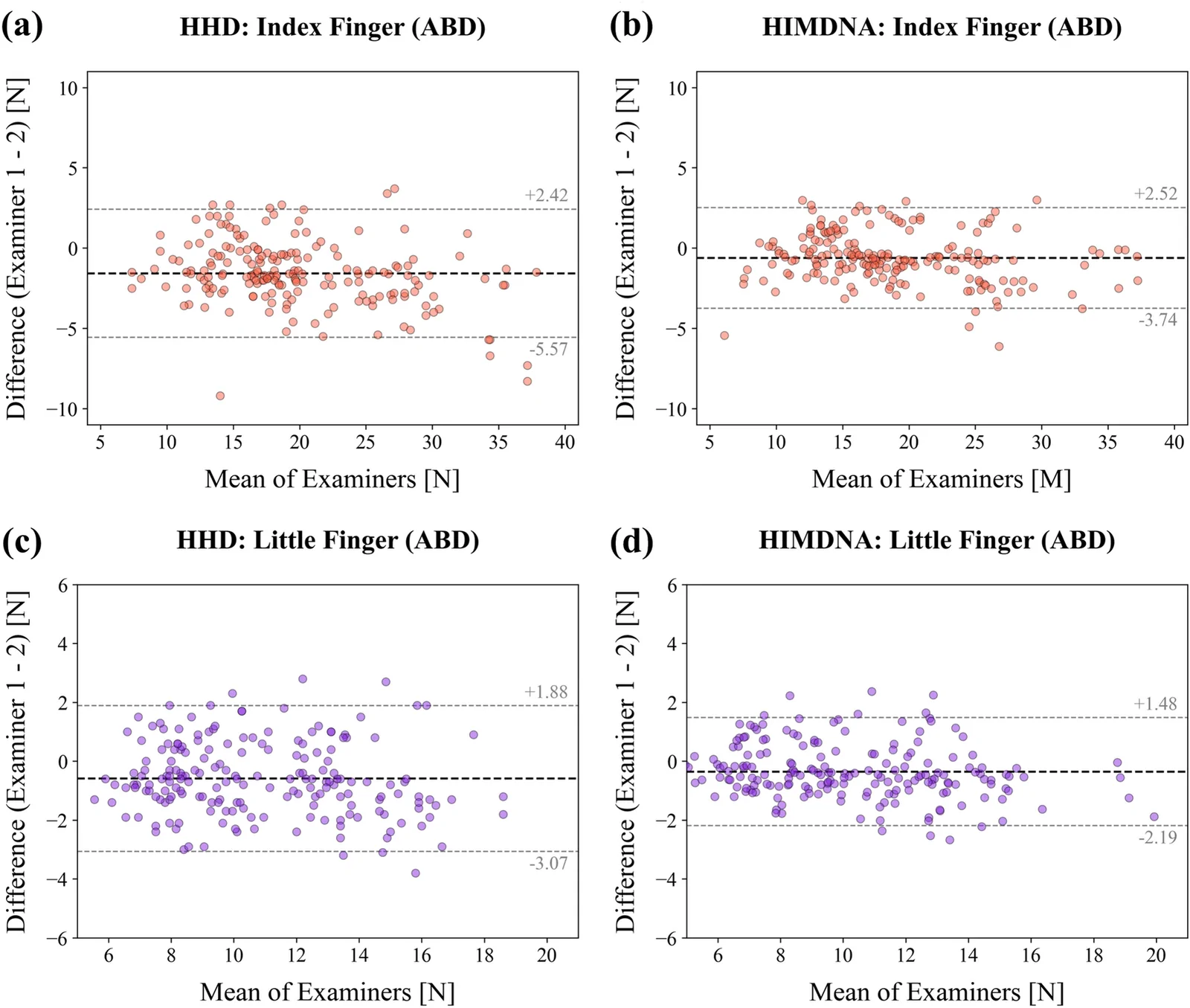
Bland–Altman analyses of inter-rater reliability for **a**, **b** index finger abduction measurements and **c**, **d** little finger abduction measurements. ABD, abduction

The inter-rater reliabilities of HIMDNA and HHD are summarized in Table 4 and visualized in Figs. 4, 5. Both devices demonstrated excellent inter-rater reliability (*ICC*s ≥ 0.900) and very strong inter-rater correlations (*r* > 0.900) across all finger motions measurable with each device. Notably, for the abduction of the index and little fingers, HIMDNA exhibited significantly superior inter-rater reliability compared with that demonstrated by HHD (index finger: *ICC* of 0.928 vs. 0.966, *p* < 0.001; little finger: *ICC* of 0.951 vs. 0.900, *p* < 0.001). The MDC ranged from 1.18 to 3.13 *N* (17.01% ~ 23.78%) for HIMDNA, whereas it ranged from 2.48 to 3.99 *N* (20.18% ~ 22.84%) for HHD.

**Table 4:** Inter-rater reliability of HIMDNA and HHD

Device	Finger	Motion type	Pearson’s *r* (*p*-value)	*p*-value	ICC_(2,1)_	*p*-value	SEM, N	MDC_95_, N
HHD	Index	ABD	0.958 (< 0.001)	0.063	0.928	< 0.001*	1.44 (7.28%)	3.99 (20.18%)
HIMDNA		ABD	0.971 (< 0.001)		0.966		1.13 (6.01%)	3.13 (16.65%)
		ADD	0.972 (< 0.001)	-	0.965	-	0.73 (7.95%)	2.03 (22.03%)
	Middle	Radial ABD	0.974 (< 0.001)	-	0.969	-	0.78 (6.14%)	2.17 (17.01%)
		Ulnar ABD	0.972 (< 0.001)	-	0.963	-	0.73 (8.58%)	2.02 (23.78%)
	Ring	ABD	0.963 (< 0.001)	-	0.956	-	0.43 (7.72%)	1.18 (21.40%)
		ADD	0.973 (< 0.001)	-	0.967	-	0.75 (7.91%)	2.08 (21.93%)
	Little	ADD	0.971 (< 0.001)	-	0.968	-	0.57 (7.53%)	1.59 (20.88%)
		ABD	0.958 (< 0.001)	< 0.001*	0.951	< 0.001*	0.66 (6.51%)	1.84 (18.03%)
HHD		ABD	0.917 (< 0.001)		0.900		0.89 (8.24%)	2.48 (22.84%)

*HHD,* Handheld dynamometer; *HIMDNA,* Hand interossei muscle dynamometer; *ABD,* Abduction; *ADD,* Adduction; *ICC,* Intraclass correlation coefficient; *SEM,* Standard error of measurement; *MDC,* Minimal detectable change

^*^Statistically significant difference (*p* < 0.05)

**Fig. 6:**
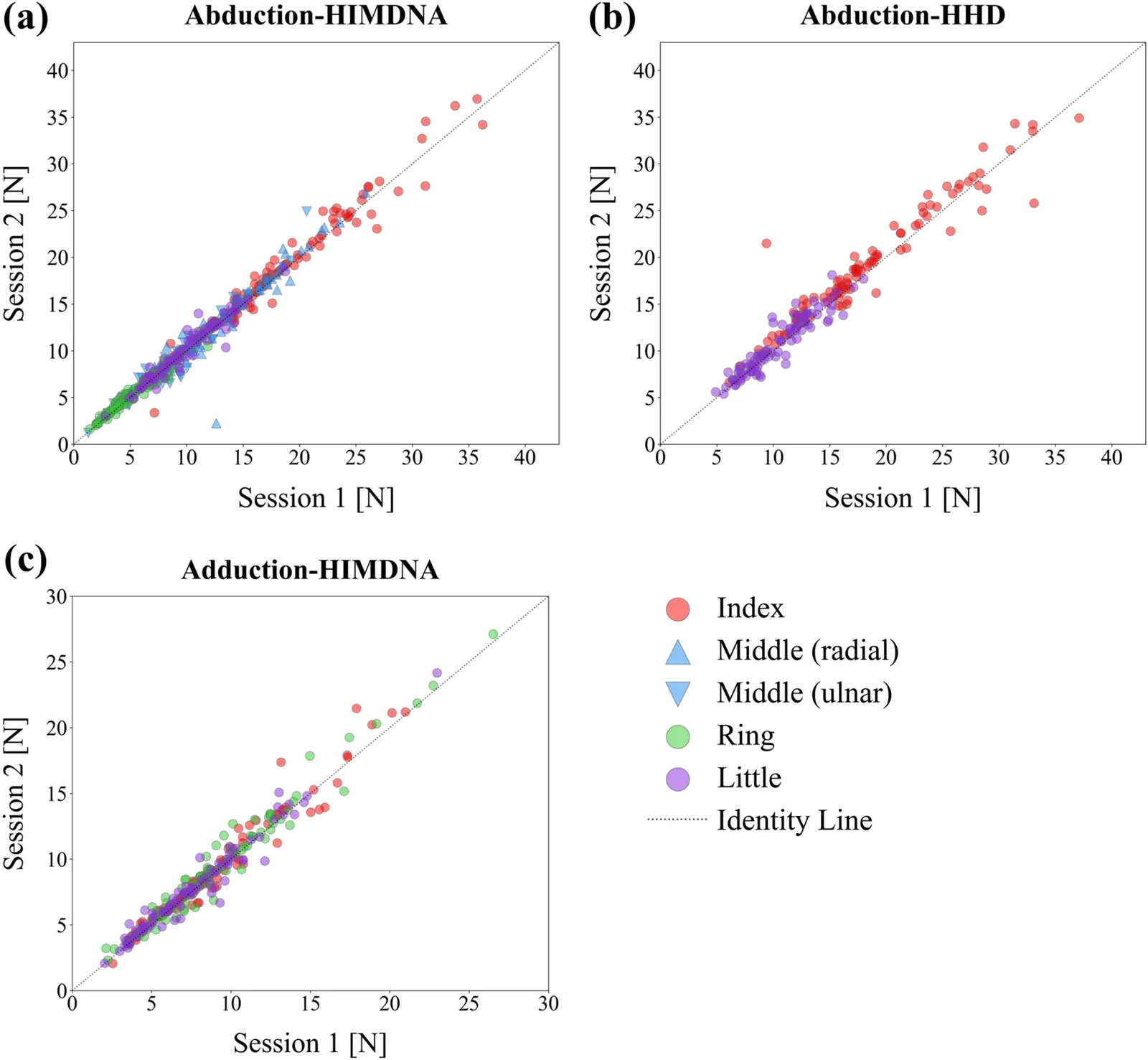
Linear regression analyses of intra-rater reliability in abduction measurements obtained with **a** HIMDNA and **b** HHD, and **c** adduction measurements with HIMDNA

**Fig. 7:**
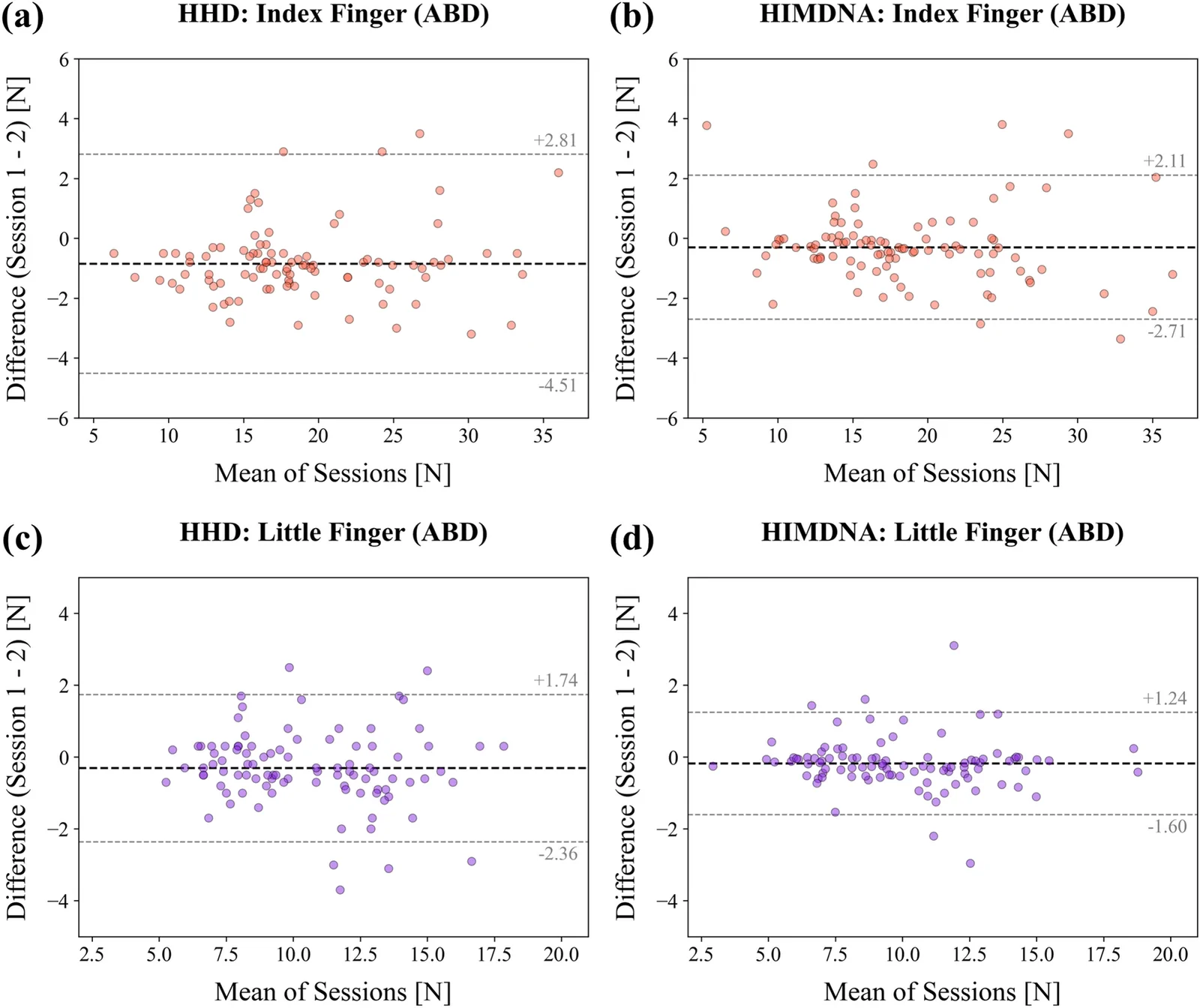
Bland–Altman analyses of intra-rater reliability for **a**, **b** index finger abduction measurements and **c**, **d** little finger abduction measurements. ABD, abduction

The intra-rater reliabilities of HIMDNA and HHD are summarized in Table 5 and visualized in Figs. 6, 7. Both devices demonstrated excellent intra-rater reliability (*ICC*s > 0.900) and very strong correlations (*r* > 0.900) across all finger motions measurable by each device. For index and little finger abduction, HIMDNA showed significantly superior intra-rater reliability compared with that exhibited by HHD (index finger: *ICC* 0.981 vs. 0.949, *p* < 0.001; little finger: *ICC* 0.971 vs. 0.935, *p* = 0.005). The MDC ranged from 0.93 to 2.57 *N* (13.31% ~ 20.53%) for HIMDNA, whereas it ranged from 2.13 to 4.01 *N* (20.18% ~ 21.09%) for HHD.

**Table 5:** Intra-rater reliability of HIMDNA and HHD

Device	Finger	Motion type	Pearson’s *r* (*p*-value)	*p -*value	ICC_(3,1)_	*p*-value	SEM, N	MDC_95_, N
HHD	Index	ABD	0.958 (< 0.001)	< 0.001*	0.949	< 0.001*	1.45 (7.61%)	4.01 (21.09%)
HIMDNA		ABD	0.982 (< 0.001)		0.981		0.89 (4.80%)	2.46 (13.31%)
		ADD	0.979 (< 0.001)	-	0.977	-	0.62 (6.91%)	1.72 (19.16%)
	Middle	Radial ABD	0.963 (< 0.001)	-	0.962	-	0.93 (7.41%)	2.57 (20.53%)
		Ulnar ABD	0.978 (< 0.001)	-	0.975	-	0.60 (7.23%)	1.66 (20.04%)
	Ring	ABD	0.980 (< 0.001)	-	0.977	-	0.34 (6.23%)	0.93 (17.26%)
		ADD	0.983 (< 0.001)	-	0.980	-	0.61 (6.62%)	1.70 (18.35%)
	Little	ADD	0.979 (< 0.001)	-	0.979	-	0.50 (6.65%)	1.38 (18.43%)
		ABD	0.973 (< 0.001)	0.007*	0.971	0.005*	0.53 (5.27%)	1.46 (14.59%)
HHD		ABD	0.940 (< 0.001)		0.935		0.77 (7.28%)	2.13 (20.18%)

*HHD,* Handheld dynamometer; *HIMDNA,* Hand interossei muscle dynamometer; *ABD,* Abduction; *ADD,* Adduction; *ICC,* Intraclass correlation coefficient; *SEM,* Standard error of measurement; *MDC,* Minimal detectable change

^*^Statistically significant difference (*p* < 0.05)

### Usability assessment

**Fig. 8:**
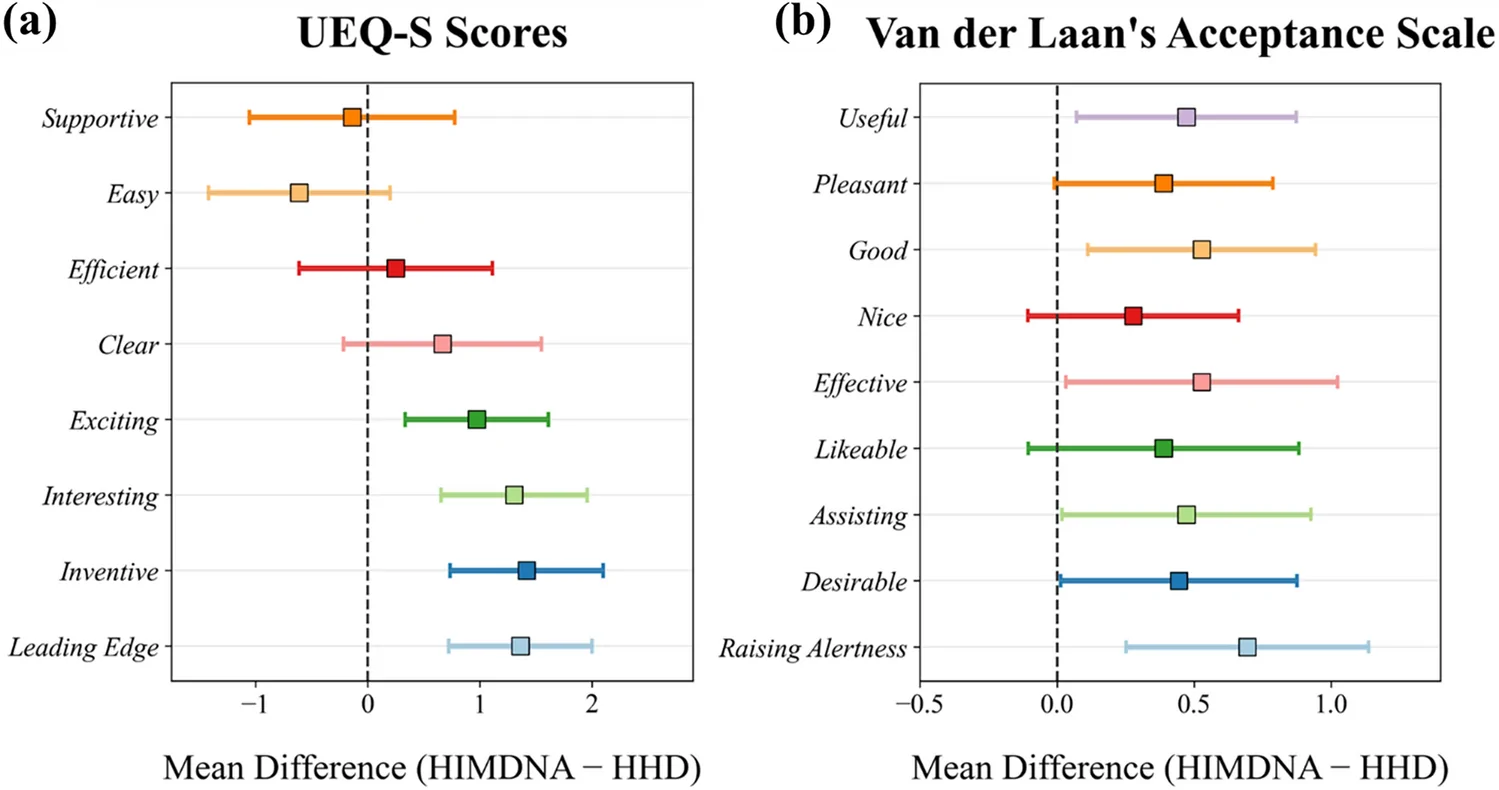
User experience survey result scores

The mean differences of usability assessment between HIMDNA and HHD are visualized in the forest plots as shown in Fig. 8. User acceptance, assessed using the Van der Laan Acceptance Scale, favored HIMDNA over HHD, with higher ratings for usefulness (*p* = 0.022), overall quality (better; *p* = 0.014), effectiveness (*p* = 0.037), assistance (*p* = 0.042), desirability (*p* = 0.044), and alertness (*p* = 0.003). By contrast, the UEQ-S showed no significant differences between the devices in terms of supportiveness, ease, efficiency, and clarity. Nevertheless, HIMDNA was perceived as more exciting (*p* = 0.004) and as more interesting, inventive, and leading edge (all *p* < 0.001).

## Discussion

In this study, a novel device called HIMDNA, designed for the quantitative assessment of finger abduction and adduction strength, was developed and validated. The design structures of HIMDNA enabled standardized and accurate measurements of both abduction and adduction from the index finger to the little finger. Because no established gold standard device exists for measuring finger abduction and adduction across multiple fingers, the conventional HHD was adopted as a practical comparator. Compared with the HHD, which can assess only the abductions of the index and little fingers, HIMDNA demonstrated strong validity, superior inter- and intra-rater reliabilities, and overall better usability. Although direct comparisons for the other finger movements, such as adduction of all fingers and abduction of the middle and ring fingers, were not feasible due to the structural limitations of HHD, the strong agreement in the reference fingers supports the reliability and accuracy of the additional motions measured by the present device.

Weakness in the hand interossei muscles frequently occurs following trauma or disease affecting the ulnar nerves [23]. Therefore, assessments of the interossei muscle strength can serve as an important indicator of disease progression or recovery. In clinical practice, the most accessible method is MMT using the MRC scale; however, this approach provides only six discrete grades and lacks objectivity and inter-rater reliability. Other bedside tests, such as Egawa’s test, Wartenberg’s sign, and little finger abduction and adduction tests, provide only binary outcomes without quantitative information [5]. Commercially available grip and pinch dynamometers provide more standardized measurements; however, both are substantially influenced by extrinsic muscle activity [18, 23]. In particular, pinch dynamometers predominantly assess the function of specific thumb-related muscles rather than interossei muscle performance. Indeed, in the present study, grip and pinch strengths showed only moderate correlations (Pearson’s *r* = 0.358 ~ 0.715) with finger abduction and adduction strength, underscoring the limitations of these traditional measures as surrogates for interossei muscle assessment. Notably, whereas index- and little finger abduction tended to correlate most strongly with grip or pinch strength, this also suggests that the remaining motions, such as finger adduction or middle and ring finger abduction, may offer unique clinical value that cannot be captured by existing methods. Moreover, normative values of finger abduction and adduction strength in healthy adults were established through this study, highlighting the potential of HIMDNA as a precise and clinically applicable tool for evaluating interossei muscle function.

Prior efforts to isolate interossei muscle strength have been limited. Schreuders et al. [23] developed the RIHM, which showed excellent reliability. They showed quantitative results for index and little finger abduction and thumb movement, but unfortunately, results for evaluation of other fingers and for adduction movements were not reported in the prior studies [26–28]. Li et al. [27] designed a device capable of quantifying multidirectional forces of the index finger, with potential applicability to other digits; however, its large and complex structure hindered its clinical feasibility. Pataky et al. [26] introduced an instrument that could measure multifinger abduction and adduction simultaneously, mimicking the spreading and squeezing movements used in daily activities. However, performing the measurements under fixed metallic slots may introduce potential errors, and their results contradicted consistent findings from other studies, including those of the present study and of Matheson et al. [44], which showed abduction forces exceeding adduction in both the index and little fingers, and radial abduction of the middle finger being stronger than ulnar abduction. Against this backdrop, the present study is the first to systematically validate the accuracy and reliability of a device capable of measuring the abduction and adduction of each finger against an established reference and to provide normative values based on the largest healthy cohort to date.

Several design features of HIMDNA contributed to its high accuracy and reliability. Among these, two aspects were particularly important. First, the load cell is mounted directly onto the force plate and integrated within a fixed structural frame. In the absence of commercially available devices specifically designed to measure finger abduction and adduction strength, a conventional HHD was employed as the reference device. However, in such measurements, the examiner must grip the device and provide a counterforce by hand, which introduces rater dependency. As a result, measurement consistency depends on how uniformly counterforce is applied because small variations in force direction, contact point, and stabilization can occur not only between examiners but also across repeated trials by the same examiner. These factors may introduce variability owing to differences in the examiner’s strength, finger positioning, and the distance between the device and the tested finger. HIMDNA eliminates these examiner-dependent factors by providing standardized positioning and eliminating the need for counterforce. This reduction in examiner-dependent variability in isometric measurements likely underlies the higher inter- and intra-rater reliability and lower MDC values observed with HIMDNA than with HHD, as reflected by the significantly greater ICC values in both index and little finger abduction. Another important design feature of HIMDNA is the incorporation of a linear guide mechanism between the opposing force plates. Earlier studies emphasized the role of such guide structures in enhancing stability by constraining movement to a single axis and ensuring accurate load transfer [45, 46]. Based on these characteristics, the proposed design directs finger forces along a single translational path, thereby minimizing off-axis loading and ensuring that the measured signal reflects only the intended abduction or adduction force.

The Lafayette HHD is limited by a minimal detectable force threshold of 5 *N*, making it unsuitable for assessing weaker interossei muscles, where subtle variations in strength may have important diagnostic implications in early-stage neuromuscular impairments [11, 47]. By contrast, HIMDNA was able to detect low-level forces below this threshold with high sensitivity. The load cell used in HIMDNA had a capacity of 20 kg with nonlinearity, hysteresis, and repeatability controlled within 0.5% of the full scale and a rated output of approximately 1.0 mV*/V*. Combined with 24-bit analog-to-digital conversion and 10 Hz sampling, the system achieved an effective resolution of approximately 0.02 *N*, allowing reliable detection of even minor changes in applied force. In fact, this sensitivity was reflected in the successful measurement of abduction or adduction forces below 5 *N* in several participants, e.g., ring finger abduction in the female participants, which was 4.5 *N* on average, thereby extending applicability to weaker interossei muscles and early-stage neuromuscular impairments.

However, despite the aforementioned improvements, several methodological considerations should be noted. First, the study population consisted solely of healthy adults in their 20 to 30* s*, which may limit the generalizability of the results and reduce their applicability to older individuals who are more prone to ulnar neuropathies such as cubital tunnel syndrome. Nonetheless, interossei hand muscle strength may not differ substantially between young and middle-aged adults and thus, the present data can still serve as a reasonable normative reference for clinical and research applications [48]. Second, HIMDNA was validated against an HHD, which served as the most appropriate available reference only because no universally accepted gold standard exists thus far for the direct measurement of finger abduction and adduction strength. Future studies incorporating other reference devices, such as RIHM, may be also valuable if the device becomes readily available. For the present study, HHD was used because it has been widely used and readily accessible in clinical environments and prior dynamometry studies [17, 34–36]. Although the HHD has known manufacturer accuracy specifications, it was used in this study as a clinical comparator rather than as a definitive reference for absolute force measurement. Therefore, the present validation should be interpreted as an assessment of agreement and measurement reliability relative to an established clinical device. Third, although the device was designed to position each finger in a certain manner, i.e., such that the PIP joint consistently contacted the plate, to standardize the point of force application, variability in hand size and finger length may have influenced the recorded forces through differences in the lever arms. Adjustable modules or size-specific configurations could reduce such variability and improve measurement accuracy across diverse hand anatomies. Lastly, measurements were obtained using a single HIMDNA unit, and inter-device reproducibility across multiple identical prototypes was not evaluated. Although it has been typical in dynamometer reliability and validity studies to assess using a single device [17, 23, 49–51], future studies examining consistency across multiple prototypes would further support clinical applications.

In conclusion, this study developed and systematically validated a novel HIMDNA designed to quantitatively measure finger abduction and adduction strength from the index finger to the little finger. The device demonstrated strong agreement with a conventional HHD, confirming its validity, and exhibited superior inter- and intra-rater reliabilities as well as overall better usability. The structural design minimizes examiner-dependent variability, provides high measurement sensitivity, and enables standardized assessment across multiple fingers. Furthermore, this study established normative values for finger abduction and adduction strength in healthy adults, offering a reference framework for future comparisons. Based on these findings taken together, HIMDNA may serve as a reliable and precise tool for the quantitative evaluation of interossei muscle strength and has potential clinical applicability on individuals with ulnar nerve disorders.

## Supplementary Information

Below is the link to the electronic supplementary material.

Supplementary file1 (PDF 132 KB)

## Data Availability

The data acquired in this study are not openly available due to the sensitive nature of human data (e.g., age, sex, height, and weight). A de-identified dataset containing the full demographic and clinometric data is available from the corresponding authors upon reasonable request.

## Material Availability

The design files and software associated are not openly available due to institutional restrictions. Additional technical information may be provided by the corresponding authors upon reasonable request.

## Code Availability

The source codes and software associated are not openly available due to institutional restrictions. Additional technical information may be provided by the corresponding authors upon reasonable request.
