# Supplementary material for: Design and Validation of a Hand Interossei Muscle Dynamometer (HIMDNA) for Finger Abduction and Adduction Strength Measurement
Source: Ann Biomed Eng. 2026 Mar 21;54(10):3669–84. doi: 10.1007/s10439-026-04077-x (PMC13649930; doi:10.1007/s10439-026-04077-x)
Supplement: Supplementary file 1 [file 10439_2026_4077_MOESM1_ESM.pdf]

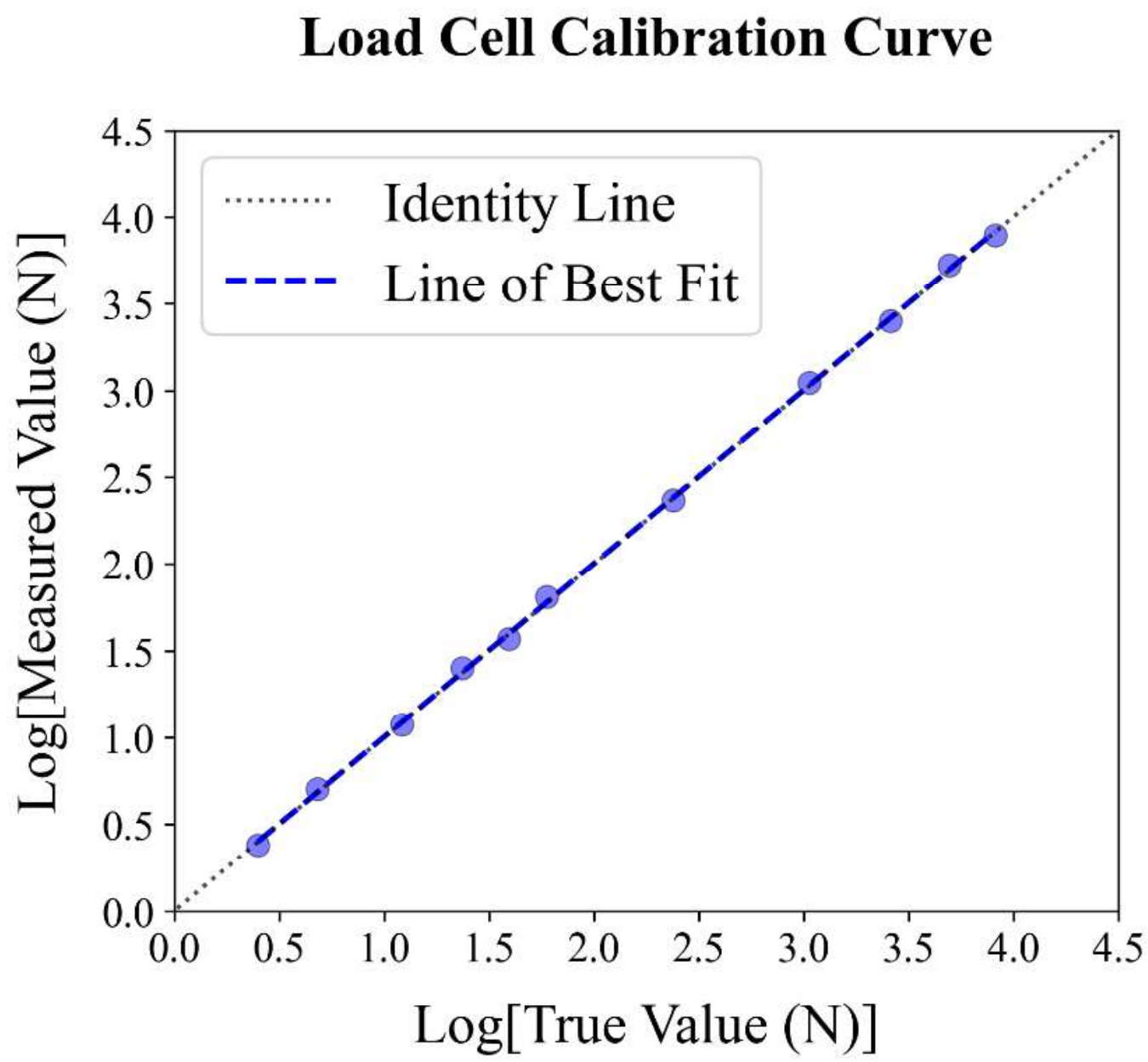

Supplementary Fig. S1 Calibration curve of the load cell in HIMDNA
